# A scoping review on the use of a participatory approach in interventions to combat overweight and obesity in low socioeconomic status communities

**DOI:** 10.1007/s00394-025-03736-4

**Published:** 2025-06-21

**Authors:** Tim van Zutphen, Claire Gaudichon, Jacub Morze, Kalliopi Anna Poulia, Gonçalo Rosas da Silva, Ascensión Marcos, Hinke Haisma

**Affiliations:** 1 Faculty Campus Fryslân, University of Groningen, Groningen, The Netherlands; 2 Université Paris-Saclay, Agro Paris Tech, INRAE, Gif-sur-Yvette, 91190 France; 3 College of Medical Sciences, SGMK Copernicus University, Ul. Nowogrodzka 47a, Warsaw, 00-695 Poland; 4 Department of Food Science & Human Nutrition, Agricultural University of Athens, Athens, 11855 Greece; 5 School of Biological Sciences, Queen’s University Belfast, Northern Ireland, Belfast, BT9 5DL UK; 6 Department of Metabolism and Nutrition, Instituto de Ciencia y Tecnología de Alimentos y Nutrición, Madrid, 28040 Spain; 7 Faculty of Spatial Sciences, Population Research Centre, University of Groningen, University of Groningen, Groningen, 9747 AD The Netherlands

**Keywords:** Participatory, Overweight, Obesity, Low socioeconomic status, Community-based participatory research

## Abstract

**Purpose:**

The burden of obesity on individuals and society has received much attention. However, most interventions to combat obesity as well as reviews focus on a (bio)medical approach. Applying an interdisciplinary approach that includes participation of the most heavily burdened low-socioeconomic status (SES) groups, are scarce. The purpose of this scoping review is to identify the characteristics and achievements of studies that applied a participatory approach to inform future development of interventions aiming to reduce obesity among low SES communities.

**Method:**

We conducted a scoping review on interventions or initiatives aiming at obesity among groups with low-SES that apply a participatory approach, i.e. that involve the target population throughout the process.

**Results:**

4246 papers were identified and screening of abstracts resulted in 37 eligible papers, resulting in 12 included papers after full-text screening. Among them, 9/12 derived from US; 7/12 were theory-based; 8/12 targeted individuals; 7/12 applied a CBPR protocol; 6/12 were participatory in the development, implementation, assessment, and evaluation phase; 4/12 applied an RCT for impact assessment and 1/12 used solely qualitative methods. 9/11 studies observed a reduction in weight/BMI.

**Discussion/Conclusion:**

Participatory approaches to inform and execute interventions to tackle obesity in low SES communities is an emerging approach. The findings show that it is feasible to co-create context-sensitive interventions, that can be beneficial on obesity outcomes, by combining theory and expertise by experience.

## Introduction

The worldwide prevalence of obesity has nearly tripled since 1975 and is considered to be an escalating pandemic. The relative increase in high body mass index (BMI) exposure was the highest over the last 30 years of all 84 risk factors included in the Global Burden of Disease Study [1]. Over 2 billion people are overweight or obese and its prevalence is not limited to high-income countries, in fact the greatest number of people with obesity live in low- and middle-income countries [2]. Despite that mostly non-communicable disease (NCD) such as cardiovascular disease (CVD), type 2 diabetes (DM2) and stroke have been linked to overweight and obesity, high BMI has also been positively associated to several types of cancers, dementia, musculoskeletal disorders and hence multimorbidity [3, 4]. Moreover, obesity is also a cause of stigma as well as psychological conditions and despite the evident link with non-communicable disease the Covid-19 pandemic highlighted the relevance of obesity in infectious diseases [5].

The shift towards chronic diseases that dominate healthcare nowadays, has also shown that the healthcare system is ill equipped for this shift and solutions are urgently needed to ensure its sustainability [6]. Despite that obesity itself was recently coined a disease, it is also a societal and economic burden that extends beyond the healthcare domain and is therefore considered to be a public health problem [7–9].

The primary foundation for obesity development is a diet with a caloric excess, possibly combined with an inactive lifestyle, resulting in energy disbalance. However, underneath these avoidable, or modifiable risk factors that also include other lifestyle aspects such as sleep and stress, a multidimensional array of interacting causes has been identified that include genetic, biological, cultural, social as well as environmental factors. The European initiative Determinants of Diet and Physical Activity even identified over 60 determinants for both dietary behavior as well as for physical activity in ethnic minority groups [10, 11].

Whereas obesity used to be associated with the more affluent part of the population and in low- income countries this is still the case, in industrialized countries this relationship has reversed, and a strong correlation has been observed between obesity and low socioeconomic status (based on education, income and occupation). In the United Kingdom for instance, children living in more deprived areas are twice as likely to develop obesity compared to the least deprived areas and this gap is growing [12]. Among the risk factors, many are local contextual factors, and these culminate at the neighborhood or community level. Low SES neighborhoods are proposed to be a highly obesogenic environment with less opportunity for physical activity and increased exposure to unhealthy food as well as its promotion, but also a more stressful environment, due to the unequal distribution of power and resources for instance, leading to differential vulnerability [13, 14].

Despite that many countries have national action plans against obesity, its prevalence has further increased and health disparities between SES groups have widened, thus these policies have been generally ineffective, especially for those that are in need the most [15–17]. In fact, some of these policies were said to do more harm than good, in particular due to their general focus on individual responsibility [18, 19]. For example, a focus on knowledge and skills is not an effective strategy to reduce inequalities as socioeconomically disadvantaged individuals have fewer economic or social resources to support behavior change. In contrast, structural initiatives could potentially decrease the gap, as they aim for environmental barriers that may be larger in low SES neighborhoods [16]. Interventions with a universal nature and trial-based strategies also tend to attract less and lose more disadvantaged participants [20, 21].

Given the multifactorial nature of health inequalities related to SES and the limited success of classical top-down interventions in these communities, tailored strategies are needed that are context-sensitive. Stigmatization, discontent and (institutional) distrust are among the underlying factors for limited success with interventions among the most vulnerable groups [22, 23]. One strategy to achieve a context-sensitive approach that may also avoid resistance from recipients, aims to include the target population in the process, i.e. through community-based participatory research. To our knowledge, such studies targeting obesity, have not been subject to review. In participatory research, ideally, stakeholders are involved in all phases of the research [24]: from the formative through the implementation and the evaluation phase to a feedback loop to adapt the intervention based on the experiences learned. With this scoping review, we aim to identify the characteristics and achievements of intervention studies that applied a participatory approach among low socioeconomic status communities as a learning exercise for future development of interventions aiming to reduce obesity.

Based on our exploratory analysis, we identify commonalities, strengths and limitations of the selected interventions with recommendation for future development of interventions to combat overweight and obesity in low-income populations. We conducted the scoping review using the guidance provided by Peters et al. (2015) [25].

## Methods

We conducted a search in Scopus, Web of Science and PubMed using search terms that relate to participatory, low socioeconomic status/ factors, community, obesity, overweight, interventions. The search string is presented in Table 1. The search was conducted in 2022, with an update in May 2025.

**Table 1:** Search string

Search string
TITLE-ABS-KEY ((participatory OR participative) W/3 research)
TITLE-ABS-KEY ((collaborative OR cooperative OR appreciative) W/2 inquiry)
TITLE-ABS-KEY ((action OR citizen) W/2 (research OR science))
TITLE-ABS (community W/2 (research OR program* OR trial OR work OR network* OR action OR process OR partner* OR involv* OR engage* OR particip* OR learning OR mobilization OR mobilisation OR practice OR development))
TITLE-ABS-KEY (socioecon om* W/3 (status OR factor* OR level* OR position) OR TITLE-ABS(employ* OR income OR wealth OR deprivat* OR deprive* OR occupation)
TITLE-ABS (“weight loss” OR “weight maintenance” OR “body weight” OR “body mass index” OR BMI OR “body composition” OR anthropometry OR “body fat” OR “fat mass” OR “fat-free mass” OR “lean body mass” OR overweight* OR obese OR obesity )
TITLE-ABS (“dietary” OR “diet” OR “nutrition” OR nutrient* OR food* OR dietician OR “food item*”)
#1 OR #2 OR #3 OR #4
#6 OR #7
#8 AND #5 AND #9

**Fig. 1:**
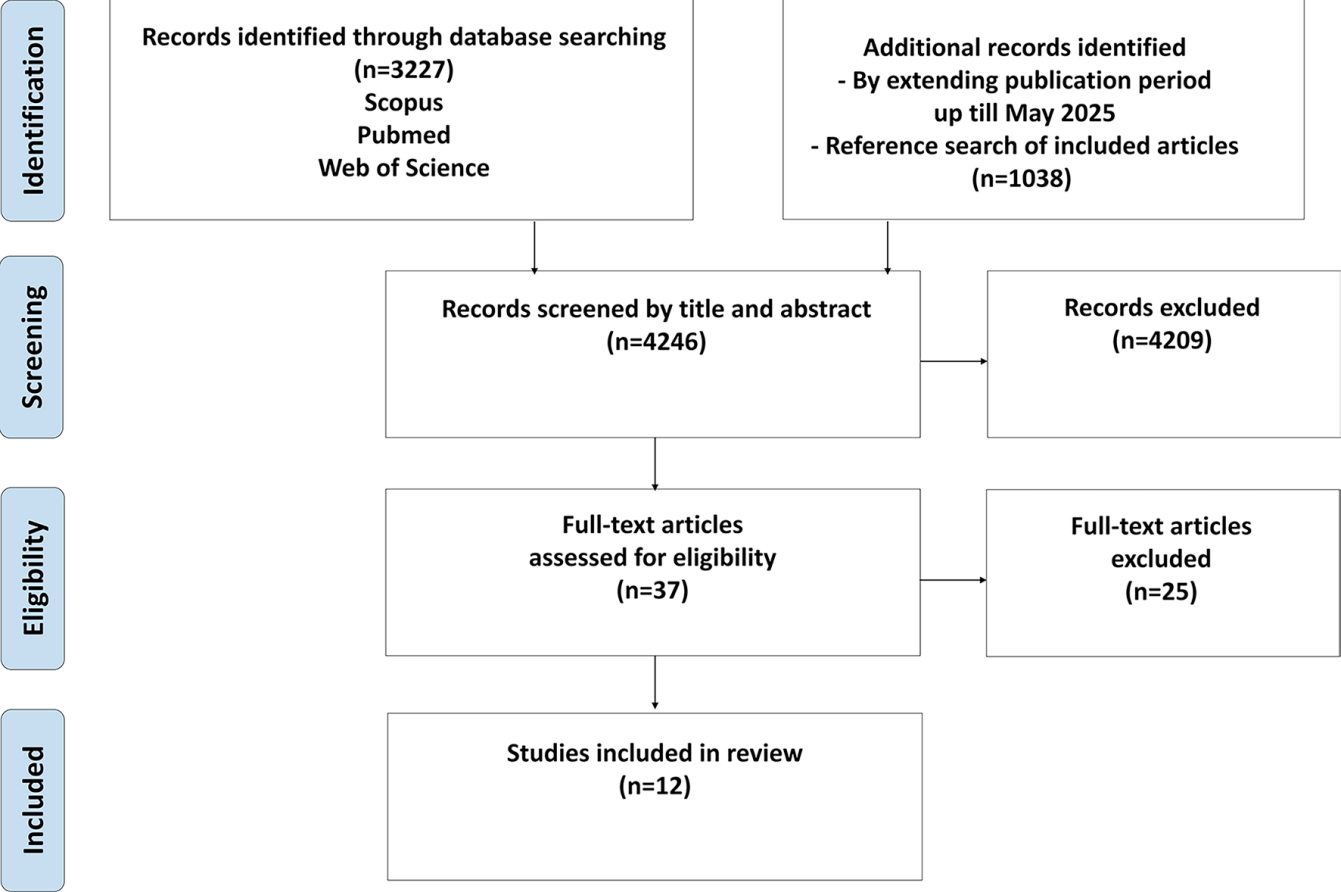
PRISMA of screened and selected papers

Selected papers were entered in Rayyan software (rayyan.ai) and the initial hits were screened by the authors of the paper in three teams, on the basis of title and abstract. Hits were classified as nor relevant, relevant, or may be relevant For “may be relevant” hits, around 10% of the total hits, cross-checks between the teams were made to reach consensus. The PRISMA of screened and selected papers is presented in Fig. 1.

## Results

### 1) Description of selected studies

The search strategy identified 4246 citations, including 84 protocols for interventions using a participatory approach. These 84 protocols were not considered for further review. Titles and abstracts were screened for meeting the inclusion criteria, yielding 36 articles. A further 12 papers were added through hand searching. Full texts were retrieved from this total set and another 36 were excluded for not meeting the inclusion criteria upon analysis of the full article, leading to a final set of 12 studies as shown from the PRISMA in Fig. 1. Mayer et al. (2019) was not found in the initial search, as this paper builds on the participatory process applied in the pilot project that was described by Parikh et al. (2010) [26, 27]. The study by Nollen et al. (2014) was preceded by an earlier paper (2013), however this earlier paper was not included as the study focused on feasibility testing of the intervention without describing impact, and did thus not meet our selection criteria [28, 29].

Included studies were published between 2008 and 2019. Nine out of 12 studies were from the United States, with single studies from Australia, Canada and India (Tables 1 and 2). Table 1 presents further characteristics of the interventions. The interventions were mostly directed towards individuals (Balagopal et al., 2012, Davison et al., 2013, Cherrington et al., 2013, Goldfinger et al., 2008, Nollen et al., 2014, Parikh et al., 2010, Reifsnider et al., 2018, Sendall et al., 2016), while one applied a systems approach that was aimed at the community (Liao et al., 2016) and two interventions targeted both the community and individuals (Wright et al., 2013, Ziabaksh et al., 2016) [27, 28, 30–38].

Below we review how the included studies made use of theory, participation and how studies were evaluated. Furthermore, the extent of participation, who participated and what approaches were used in the formative, implementation, evaluation, and feedback phase of the interventions is described in order to further classify the included studies and identify common learnings.

**Table 2:** Description of studies

Author	Location	Population	Intervention
Balagopal et al., 2012	India, rural Gujarat	Adult rural community	Community health worker-delivered educational lifestyle intervention in high- and low SES groups.
Cherrington et al., 2015	USA, Birmingham, Alabama	Overweight Latina immigrants	Promotora-led interactive healthy lifestyle quasi-experimental pilot study
Davison et al., 2013	USA, upstate New York	2–5 year old children	Parent-led intervention aiming at nutritional as well as communication, and social skills
Goldfinger et al., 2008	USA, Harlem, New York	African American adults	Peer-led educational program to promote healthy lifestyle and reduce weight
Liao et al., 2016	USA, 10 states	14 black communities	Large 4-year place-based systems approach intervention in 14 communities on obesity
Mayer et al., 2019	USA, Harlem, New York	Overweight/ obese prediabetic adults	Peer-led workshop series to promote healthy lifestyle RCT
Nollen et al., 2014	USA, Kansas City	9–14 year old ethnic minority girls	Randomized pilot trial, mobile technology-based dietary behavior stimulation
Parikh et al., 2010	USA, Harlem, New York	Adults with prediabetes	Randomized pilot, peer-led diabetes prevention education program
Reifsnider et al., 2018	USA, southwestern metropolitan area	Infants of Mexican-American women	Promotora-led parent counseling on healthy infant growth and development RCT
Sendall et al., 2016	Australia, southeast Queensland	Truck drivers	Workplace health promotion intervention
Wright et al., 2013	USA, Los Angeles	8–12 year old school kids	Nurse-led family-centered lifestyle intervention RCT
Ziabakhsh et al., 2016	Canada, British Columbia	First nations indigenous women	Nurse & indigenous cultural lead-led broad wellness and heart health promotion pilot

#### Use of theory

Seven interventions were theory-based. Five of them applied theories from social psychology: self-efficacy (Parikh et al., 2010; Mayer et al., 2019) / self-determination theory (Cherrington et al., 2015)/ the principles of behavioural weight control (Nollen et al., 2014); the family ecological model (FEM) - a family centered developmental theory that emphasizes the context as a shaping factor for family behaviour - as well as empowerment theory a framework aimed at enhancing self-determination of individuals and communities (Davison et al., 2013) [26–28, 31, 32]. In the FEM, caregiving practices and family daily living strategies are shaped by factors that are proximal to families in combination with their broader contexts. Nollen et al., (2013,2014) applied grounded theory, a theory that derives concepts from the data and develops them by collecting, coding and analysing data concurrently [28, 29]. Finally, Ziabakhsh et al. (2016) applied feminist theory [38].

#### Use of participatory protocol

Studies used a range of protocols for their participatory research. Of the 12 studies selected, seven used community-based participatory research (CBPR) [26, 27, 30, 31, 33, 36, 37] using the guidelines for such research as described by Peters et al. (2015) [21]; two studies in Latino communities in the USA involved promotora’s, i.e. community members without formal healthcare education but often with specialized training to liaise between communities and health and social service providers [32, 34]; two studies applied a community-engaged approach [28, 38]) and one study applied PAR (participatory action research [35]).

Studies claiming to apply CBPR without detailing its implementation entirely were excluded during the screening process.

#### Study assessment design

The study design included a control group in half of the studies ( [26–28, 30, 34, 36, 37] and this was by means of an RCT in all but two of these studies. In contrast, a pre-post design was applied in the remainder of the quantitative evaluations, and Ziabaksh’set al., (2016 [38]), evaluation was based on subjective assessment of health.

#### Level of evaluation

The group of interventions targeted at the *individual* included health education messages, courses and workshops that mostly addressed nutrition, physical activity and stress management that were culturally sensitive (Table 3). In contrast, the REACH US project took a *systems approach* where they targeted the “upstream” causes of health disparities in communities [36]. REACH US was launched in 2007 by the Centers for Disease Control and Prevention. Through environmental and system improvements the project facilitated healthy eating and active living by making such choices easier, more convenient, affordable, safe and a behaviour norm. This intervention stands out for its approach, its scale (14 Black communities in 10 US states), and for the duration of the evaluation (4 years of monitoring). In all these communities, the interventions were focusing on: building strong community-based coalitions; focusing on policy, systems and environmental improvements; and culturally tailored interventions. Of the two studies targeting both *communities and individuals*, Wright et al. (2013) provided weekly physical activity and nutrition education through a school-based programme (Kids N Fitness**©**) for parents and children [37]. This programme was embedded into a more integral approach and intervention sites also participated in school-wide wellness activities, including health and counseling services, staff professional development in health promotion, parental education newsletters, and wellness policies for the provision of healthy foods at the school. The second intervention targeting communities and individuals by Ziabaksh et al. (2016) aimed at reducing risk factors for cardiovascular diseases and the intervention evolved over time [38]. In their study, initially, nurse practitioners felt strongly about goal setting as a separate component of the intervention but during the intervention the Talking Circle became the backbone of the intervention into which goal setting was embedded in a culturally sensitive and appropriate way [38]. Health messages were adopted to spiritual practices of the community. For example, smoking could not simply be a risk factor as “holy smoke” is part of healing practices in the community.

**Table 3:** Study characteristics

Study characteristic	Predominant characteristic	Range of characteristics
Country	9/12 USA	USA (9)/ Canada (1)/ Australia (1)/ India (1)
Theory-based	7/12	Self-efficacy/ determination Theory/ Behavioural weight control (4)
		Family Ecological Model and Empowerment Theory (1)
		Feminist Theory (1)
Participatory protocol	7/12 CBPR	CBPR (7)
		Promotora-led (2)
		Community engaged approach (2)
		PAR (1)
Collaboration	8/12 Partnership/coalition	Partnership (6)
		Coalition (2)
		Community advisory board (4)Individual (9)
Level of evaluation	10/12 individual	Community (1)
		Both (2)
RCT	4/12	With control group (1)
Pre-post design	6/12	

Abbreviations. CBPR: Community-based participatory research, PAR: participatory action research, RCT: Randomized controlled trial

### 2) Extent of participation in intervention

To further specify the extent of participation, we ranked the selected papers based on their participative elements in the formative, implementation, evaluation, and feedback phase of the research project and categorised them into two groups. Table 4 presents participation in each of the phases of research. The first group includes studies that are participative in all phases of the research (identified as fully participatory in Table 4), while the second group is participative in only some of the phases or lacks a detailed description of the nature of the participation (identified as participatory in Table 4). For example, some studies in the second category described co-creation for the planning and execution phase, yet no clear description of collaboration in other phases such as the design was evident.

**Table 4:** Participants in various phases of selected studies

	Formative phase	Implementation phase	Assessment	Evaluation
Fully participatory studies				
Balagopal et al., 2012 - Head start [30]	Community meetings, block spokesman (village matrix)	Community health workers (CHW) as change agents	Adults (18+) from a rural community in Gujarat	CHWs and project coordinator
Davison et al., 2013 - Community for Healthy Living [31]	CAB (parents, community organization representatives, key agency Head Start staff)	Parents	Parents and children	CAB including Head Start families
Goldfinger et al., 2008– HEAL [33]	Community-academic coalition (local nutritionists, health professionals, outreach workers)	Coalition Subcommittee (local nutritionists, health professionals, outreach workers)	Peer-leaders and church members in East-Harlem	Trained research assistants and church members
Parikh et al., 2010– HEED [27]	Community-academic partnership (5 subcommittees); Community Engagement Subcommittee	Intervention Subcommittee; Latino Education Committee reviewed all study materials, Clinical Education Subcommittee developed tool kit for clinicians.	Adults in East-Harlem	Evaluation/Policy Subcommittee (Board members, Community co-investigator, East-Harlem adults)
Mayer et al., 2019 [26]	Intervention committee comprised of East Harlem residents with prediabetes, community leaders, physicians, social workers, nutritionists, DPP-involved faculty, and health educators	Pairs of peer leaders with similar socioeconomic backgrounds and health problems as the participants led the groups	Adults with prediabetes	
Sendall et al., 2016 [35]	Project team, truck drivers, and workplace managers	Selection was made by workplace managers	Truck drivers and workplace managers.	Workplace managers
Ziabakhsh et al., 2016 - Seven Sisters [38]	Indigenous women leaders and Elders as champions of heart health (BC Women’s Hospital & Health Centre in partnership with a nonprofit indigenous women’s health organization, Pacific Association of First Nations Women).	8 indigenous female leaders (formerly working as health advocates in the community) and/or as Elders.	8 indigenous female leaders; one-on-one sessions with NPs and Cultural Leaders during programme implementation	8 indigenous female leaders (participants of the study)
Participatory studies				
Cherrington et al., 2015 - ESENCIAL para vivir [32]	AB including community members, promotoras, an endocrinologist, a bicultural nutritionist, a representative from the Minority Health department’s Office and a behavioural scientist with expertise in community-based methods and Latino health	promotora-led intervention, Latino women and their spouses and family	Overweight Latina immigrants	
Liao et al., 2016 - REACH US [36]	Community-based coalitions (community-based organizations, local health depts, universities, organizations or groups with primary missions unrelated to health, such as faith-based groups, YMCA, and volunteer groups. Community members.	As in formative phase. Black leaders also served as catalysts for change and ensure that interventions were culturally appropriate and tailored to the target population’s health literacy level.	Black communities in USA	
Nollen et al., 2014 [28]	AB (identifying themes for intervention strategies) and SAB (15 girl students) for PDA development focused on Fruit and vegetable intake (From Nollen et al., 2012)	Staff at after school programmes	Racial/ ethnic minority girls (9–14 years) in after school programmes	
Reifsnider et al., 2018 [34]	AB, consisting of community leaders, WIC staff, and mothers enrolled in WIC	Promotoras trained on research procedures, child development, breastfeeding support, nutrition, parenting, safety, and sleep hygiene.	Latina mothers and infant dyads	
Wright et al., 2013 - Kids N Fitness [37]	Collaborative partnership established in 8 years between University of California and LA-based underserved communities. The Lifestyle intervention is previously developed (Monzavi et al. 2006) while the environmental part developed by a team of professionals was reviewed by a CAB composed of 14 active stakeholders (incl. academicians, school administrators, teachers and parents, and parent association members) and pretested with 25 youth who provided review and modifications.	Promotoras trained on research procedures, child development, breastfeeding support, nutrition, parenting, safety, and sleep hygiene.	preadolescents, low-income, urban children, predominantly, Mexican-American (8.3 +/- 1.6 years)	

Next, the four phases of the intervention of the identified studies are described. More specifically, the partnerships that were established as well as the methods through which these partnerships were built in the formative phase are presented; this is followed by the elements of the intervention used in the implementation phase, and the study design applied in the evaluation phase as well as impact achieved; lastly, the reflection phase describes how the learning from the process as well as the evaluation were used to continue after the intervention was finished. Table 3 describes the participants included in the various phases, while Table 4 presents the methods used.

The *formative phase* of the research includes the development of the intervention. Studies built the development of their interventions on engagement with different partnerships, such as a collaborative or community academic partnership or coalition [26, 27, 30, 33, 37]), a (community) advisory board (CAB) [28, 31, 32, 34]) and community leader-led [38]. Table 4 presents the participants of each of those coalitions/ partnerships/ advisory boards in the formative phase. The study by Wright et al. (2013) deserves some special mention [37]. Although the partnership between university of California and the underserved community was established over an 8 year period, the Kids N Fitness lifestyle intervention programme was developed previously by a university professor; only the lifestyle intervention was developed with CAB and implemented involving the School Advisory Health Council. Thus, only the environmental component of the intervention can be considered participatory.

In the *implementation phase* the participants include the change agents that would be leading the implementation of the intervention, for example parents in the case of studies in children [31] or CHWs [30], or community leaders (for example [36]), or promotoras [32, 34] or a subcommittee of the partnerships/ coalitions [27, 33]. The elements of the intervention were depending on the aim of the intervention and are presented in Table 5. This could for example include physical encounters, recipe contests, health education campaigns or Ayurvedic teaching. Such activities could be geared towards improved nutrition outcomes, physical activity, wellbeing, but could also include social or environmental elements.

**Table 5:** Methods & elements used across stages of selected studies

	Formative phase	Implementation phase	Assessment	Evaluation
Highly participatory studies				
Balagopal et al., 2012 - Head Start	8 pre-planning meetings	Face-to-face encounters with participants, Recipe contests, Ayurvedic teaching (daily yoga and meditation)	Baseline versus post intervention for total population and by SES	Responsive, open inquiry
Davison et al., 2013 - Communities for Healthy Living	25 CAB meetings in 2 years, community assessment (self-reported surveys, focus groups, photovoice, windshield surveys), assessment of child weight, dietary intake and physical activity	Health communication campaign, revised BMI letters, family nutrition counseling, parents connect for healthy living programme, child programme.	Pre-post cohort design	CAB meetings continued and meeting with parents
Goldfinger et al., 2008 - HEAL	Local survey and focus group data and coalition subcommittee experiences to identify locally appropriate diet and exercise messages from existing curricula and consulting with experts.	A Portion control and diet composition course, drinking calorie-free beverages, cutting fat, making daily life more active, eating healthy food on a budget and at fast food venues.	Baseline versus follow-up at 10 weeks, after 8th and final session of the course, and at 22 and 232 weeks and 1 year. Change in weight	Post-intervention survey
Parikh et al., 2010 - HEED	CAP; 5 subcommittees to develop a community-driven, culturally appropriate, scientifically sound diabetes prevention intervention. Community Engagement Subcommittee developed the intervention	Modification of HEAL: 8 workshops: Diabetes prevention, healthy foods access, label reading, fun physical activity, planning a healthy plate, making traditional foods healthy, and portion control.	Randomized intervention/ delayed intervention. Validated scales to assess KAB, validated FFQ, Global Physical Activity Quest.	Interviews and focus groups
Mayer et al., 2019	A committee of East Harlem residents with prediabetes, community leaders, physicians, social workers, nutritionists, DPP-involved faculty, and health educators developed the curriculum tailored to meet the needs of a low income population	Modification of HEAL and HEED using peer-led workshops for affordable healthy cooking, daily physical activity, coping with stress, buddy system.	RCT with controls on a waitlist for intervention one year later	
Sendall	One-to-one interviews on physical activity and healthy eating, focusgroups and truck drivers’ health, healthy eating and p.a., perceptions about health at work.	Intervention components: Healthy eating posters; healthy options vending machine, supply of free fruit, 10000 steps workplace challenge, toolbox talks, health messages (in e.g. pay slips), Trucking’ Healthy Facebook webpage.	Pre-post-final evaluation, qualitative findings are presented elsewhere, paper -based surveys.	Feedback to workplaces for intervention design
Ziabakhsh et al., 2016 - Seven Sisters	A contextualised group-based health promotion programme (focused on cardio-vascular disease)	Women-only group sessions for 8 weeks, including Sacred Blanket ceremony, Talking Circle breakfast and educational component, weekly assignments and gifting;.	Questionnaire on diet, physical activity, and smoking with additional focus on emotional and psychological factors. One-on-one sessions to confirm the themes identified.	FGD, evaluation framework developed by providers, programme planners, community partners
Participatory studies				
Cherrington et al., 2015 - ESENCIAL para vivir	Quarterly meetings of the advisory board; community member focus groups and 18 semi-structured interviews with managers and promotora to develop intervention themes; motivational interview technique training for promotoras	Groups and individual sessions centered on personal and family-level values related to health & wellbeing, and sessions on: diabetes prevention, healthy living barriers, healthy diet, physical activity, stress, autonomy, competence and relatedness to others.	Baseline, after 8 week intervention, and 6 mo follow-up with historical control. Physiological outcomes and scales for behaviour.	NA Maintenance sessions were deemed beyond the scope of the intervention and evaluation of the findings are not discussed
Liao et al., 2016 - REACH US	Community based coalitions; Focusing on policy, systems, and environmental improvements; culturally tailored interventions.	Structurally improving the obesogenic environment: limiting new fast-food restaurants, expanding healthy food options. Creation of neighbourhood farmers’ markets and gardens. Implement policies and infrastructure to support physical activity. Worksite wellness policies and revitalisation of community environment Community-wide health promotion.	Annual cross-sectional surveys/ comparison with BRFSS (Behavioural Risk Factor surveillance System)	Not specified for the 14 community interventions combined
Nollen et al., 2014	Phase I: two focus groups with girls evaluated with grounded theory; Phase II: PDA with five components: education and feedback on diet, goals setting/reminders/completion, encouragement and problem solving. Phase III: Adding components: Morning general reminder; food diary; nightly feedback and goal planning, Reward system, health education. (detailed in Nollen 2013)		Randomised-pilot study: goal setting, reward systems, quant evaluation of FV, SBB and screen time.	
Reifsnider et al., 2018	unclear/ not described	One prenatal and seven postpartum home visits during the first year to discuss infants’ growth, health, development, sleep and play/exercise activities. Also, lactation consultation if needed	RCT, one-week post-partum, infant age 1, 6, and 12 months	
Wright et al., 2013 - Kids N Fitness	Quarterly meetings of CAB to advise researchers on all phases of the study design, recruitment, retention, and dissemination of information	A fixed family-centered educational lifestyle program, described by Monzavi et al., 2006. School-level environmental activities involving wellness policy with dietary changes and staff development. The community establised partnerships with local clinics for health and mental health services.	RCT (parallel group) with intent to treat analysis, baseline, 4 and 12 months	

CHW - community health workers

CAB - community advisory board

SAB - student advisory board

Participants in the *evaluation phase* include the target population as well as those who did the assessments, although most studies did not specify the latter. Impact of the interventions was assessed based on individual-level or community-level indicators. The individual-level interventions used outcome indicators in the physical domain, such as anthropometric indicators, blood pressure, fasting glucose but also behavioural indicators such as dietary intake or food practices, knowledge scores and attitudes. Most studies described above used quantitative measures to assess impact. In contrast, in an indigenous population in Canada, Ziabaksh et al. (2016) applied a qualitative approach and focused on small steps achieved on dietary patterns, physical activity, and emotional as well as spiritual health [38]. The large-scale study by Liao et al. (2016) was evaluated based on the assessment of prevalence of obesity in the REACH US populations as compared to propensity matched controls from the Behavioural Risk Factor Surveillance System [36]. Interestingly, almost all studies described a positive effect of the intervention on the outcome indicators, and Davison et al. even found a dose-response effect of the intervention [31]. Only the interventions directed at parents of infants by Reifsnider et al. (2018) did not show an effect on outcome indicators, including overweight/ obesity status at age 12 months [34]. Nollen et al. (2014) observed only trends towards increased food and vegetable consumption as well as decreased sugar-sweetened beverage consumption of girls in a mobile device intervention [28]. In contrast, the school-based study by Wright et al., (2013) demonstrated a difference in effect on BMI reduction between boys and girls and pointed towards the importance of a gendered approach to prevent health disparities (Table 4) [37].

The final *reflection phase* of the interventions distinguished the two categories (highly participatory and participatory together with the formative phase. The reflection phase involved a reflective meeting, focus groups or a survey with the target population and/ or with the change agents and/ or coalition.

#### Examples of studies with full participation throughout the research project

To further illustrate the nature of the participation, two studies from the first category are described.

Davison et al., (2013) aimed to reduce childhood obesity together with families in upstate New York during a one-year multifactorial family-centered intervention [31]. The Family Ecological Model and Empowerment theory were used as theoretical underpinnings for the development of the intervention. Parents played an active and equal role in community assessment and using the findings to design a family-centered childhood obesity intervention. Parents also played a leading role in implementing the intervention and worked with the research team to evaluate the findings. The intervention included a poster campaign, BMI feedback letters and counseling sessions for parents as well as a program aimed at the children. A pre-post design at baseline and after a year was applied for evaluation [31].

The only qualitative study identified, concerns the development of a women-centered and culturally responsive heart health promotion programme among indigenous women in Canada by Ziabakshsh et al. (2016) [38]. The Seven Sisters intervention was informed by indigenous healing perspectives, transcultural nursing, and feminist theories of health and illness. This project engaged indigenous women leaders and Elders as champions of heart health who would simultaneously learn about and try to improve their own personal risk factors while contributing to shaping the healthy living practices of their community members. This approach reflects the indigenous value that wisdom comes from Elders and leaders. The Talking Circle that initially had been included as a minor part of the intervention, became more and more relevant in the course of the programme and other items, such as goal setting were eventually embedded into the culturally accepted Talking Circle. Evaluation involved analysis of the Talking Circle’s contents, a focus group, field observations, and self-completed surveys.

#### 3. Impact of the interventions

Although a scoping review is not designed to evaluate (the direction of) impact, the selected studies broadly show positive outcomes in BMI/obesity reduction and other health indicators, across all fully participatory studies with quantitative approaches, independently of the demographics of the study populations (Table 6). Most notably, Mayer et al. (2019) and Liao et al. (2016) [26, 36] both showed a marked reduction in body weight and obesity respectively in relation to their established control groups, which is significant due to both the design and participant number in these studies, especially in the latter. Results in the studies with a lower level of participation were more mixed, and two of the four studies (Nollen et al., 2014 and Reifsnider et al., 2018 [28, 34]), showed no significant improvements in the measured parameters. However, the added value of a participatory approach should not be extrapolated from these results alone, as the studies herein differ significantly in aim, design and population demographics. Direct comparisons between each intervention and suitably-matched study would be required for adequate impact assessment.

**Table 6:** Study design, outcome measures and impact

Author	Design	Nutrition	Physical activity	Other	BMI/Weight
Balagopal et al., 2012	6 months *N* = 764#	F: + 0.03. V: + 0.13	+ 9.3%	− 0.9 mg/dL fasting blood glucose. -7 mm Hg systolic blood pressure	− 1% obesity
Davison et al., 2013	1 year *N* = 423	F: -0.3. V: 0 E: -189 kcal	+ 19/10 min light/moderate activity	− 61 min screen time	− 0.14 BMI Z-score. Obesity − 4.5%
Goldfinger et al., 2008	10 wks + 1 year follow-up, *N* = 26	F: + 0.7. V: + 0.7. Fat − 7 g	No change - 1 h sedentary behavior	+ perceived quality of life	− 2.2 kg at 10 weeks. - 4.9 kg at 1 yr
Parikh et al., 2010	10 weeks + 1 year follow-up. *N* = 50	F: 0. V: 0	No change	Qualitative improvements in lifestyle & empowerment	− 3.6 kg at 1 year follow-up
Mayer et al., 2019	6 months. *N* = 210	F: 0. V: 0	No change (text says less sedentary behavior)	- Diabetes development probability	− 1.4 kg at 6 months vs. -0.5 in controls
Liao et al., 2016	4 years. *N* = 8765	NR	NR	NR	− 2.1% obesity, − 3% relative to control in 3 yrs
Sendall et al., 2016	6 months. *N* = 22$	F: + 23%. V: + 21%	− 26% all-day sedentary behavior	+ 11% good self-reported health	− 16% BMI above 30$$
Ziabakhsh et al., 2016	8 weeks *N* = 8	F: + V: + - Processed food	More conscious of need to be active	Qualitative approach	NR
Cherrington et al., 2015	8 weeks, 6 months follow-up *N* = 22	E: -486 kcal + 3% calories from protein	+ 47 min moderate/vigorous activity (but accelerometer not)	+ 50% no depressive symptoms	− 2.2 kg, -0.9 BMI at 8 wks. Not sustained
Nollen et al., 2014	4–12 weeks@. *N* = 26	F: +1 (trend). V: +1 (trend). SSB: -0.35 (trend)	No change in screen time	Response to cues associates with SSB	No change
Reifsnider et al., 2018	1 year *N* = 119	NR	NR	Overweight/obesity more common in formula-fed infants	No change in BMI z-score
Wright et al., 2013	6 weeks, 1 year follow-up, *N* = 251	NR	+ >60 min/day activity participation. Decreased tv viewing	No change in computer/video game use	− 0.3 BMI in girls, - same trend in boys

F = servings of fruit, V = servings of vegetable, E = energy kcal/day, SSB = sugar sweetened beverages, NR = Not reported, * The presented sample size is the size of the intervention group, # Balagopal reports 1638 participants of which 764 in the low SES group, $ 46 pre-test, 22 post-test, $$ self-reported BMI, @ F&V outcomes at 4 weeks, SSB at 8 weeks, screen time at 12 weeks

## Conclusion & discussion

Participatory approaches for the development and assessment of interventions to combat overweight and obesity in low SES communities have been reported only after 2012 but not after 2019. This in itself is an interesting and surprising finding. It shows how research approaches emerge and disappear over time. The large number of protocols for participatory approaches, however, does suggest that there is still a potential for future findings on this topic. Most studies originate from the USA. CBPR was also first developed there. Given that overweight and obesity as well as associated chronic diseases are of major public health concern in the USA, this dominance of studies from the US is not surprising. However, the absence of participatory studies from the UK or Ireland, two other countries with high prevalence of overweight and obesity, is surprising, nevertheless it is an emerging approach. The findings show that it is feasible to co-create context-sensitive interventions by combining theory and expertise by experience. Stakeholder participation in the studies is formalised in partnerships or advisory boards. Description of the intervention reflection phase was only included in half of the selected studies and the evaluation approach applied is heterogeneous. Studies that omitted the reflection phase were also the ones that applied the classical biomedical RCT to assess impact. Finally, and with some caution, we conclude that interventions of sufficient length can help to reduce overweight and obesity in low SES populations and contribute to more equal health for all. For infant overweight and obesity, we cannot make a conclusion as only one study involved parents of infants and found no effect.

We applied a scoping review with a systematic search rather than a systematic review as we wanted to emphasize the explorative nature of our search. Rather than aiming to be able to make a substantial contribution to the question whether participatory approaches result in higher impact than top-down interventions, in a systematic review, it was our goal to describe the studies that we identified and try to learn from their design and analysis [21]. From this analysis, we can draw several learnings: (1) a culturally sensitive attitude by healthcare professionals and researchers can contribute to the development of an intervention that embraces local practices and beliefs and builds and maintains trust with the target population. (2) Collaboration between researchers and low SES communities as equals is possible in developing effective interventions and leads to empowerment of the target population (3) Best process versus best practices: standardize the process not the practice [31]; (4) length of study is often judged on length of intervention only, however, length of formative phase may add to impact and should also be considered (i.e [32]. (5) Participatory approaches have potential to generate sustainable impact beyond the initial timeframe of the intervention.

Our search and analysis have some strengths and some limitations. As we aimed to identify interventions in which impact was also assessed, we missed out on qualitative studies that often do not focus on impact assessment. In particular for the formative and implementation phase much can be learned from participatory approaches using a qualitative study design. Only one of the selected studies used a qualitative methodology from the formative to the reflection phase [38]. For other interventions described here, in some cases qualitative assessments were reported separately [39, 40], while no mixed methods approaches were reported. Our search did not for example, identify the EPODE studies. This may seem surprising but can be explained by the highly relevant difference between merely community-based and actual participatory research and we consider this a strength of our search and review. Where EPODE and many studies in Europe following this study as an example (for example JOGG in the Netherlands) is community-based, it does not engage with the target population to develop the intervention in collaboration [41]. Furthermore, several studies noted an effect on body weight or BMI, while dietary assessment was not affected [26, 27, 33], indicating that energy intake and energy expenditure remain difficult to assess over an extended period of time [42, 43].

The use of low SES as a search term may introduce a stigmatised lens [44]. A preferred way of addressing this population is by sticking to the actual facts, such as low income; such terms were also part of our search string in order to accommodate more sensitive descriptions of the target population. In addition, obesity stigmatization is abundant, a persistent issue that is driven by framing obesity as a personal responsibility rather than the result of a complex network of many factors including stigmatization [45]. Collective actions that include the target population from the start, instead of excluding them, may overcome this issue. Complex system approaches of the multifactorial issue of obesity in low SES communities also advocate for participation of the target population to not only inform the intervention about its particular context that could perhaps be assessed objectively to some extent but also to include experiences and perceptions of the most important stakeholder, the people themselves [46].
